# Factors affecting dysbiosis of the gut microbiota in the elderly and the progress of interventions in traditional Chinese and Western medicine

**DOI:** 10.3389/fcimb.2025.1529347

**Published:** 2025-03-24

**Authors:** Zhi-Jun Zhang, Ru Gao, Yu-Tong Lu, Zhi-Liang Zuo, Yu-Huan Li, Shan Liu, Si-Yuan Song, Yi Wang, Hongyan Lai

**Affiliations:** ^1^ Hepatological surgery department, The People’s Hospital of Wenjiang Chengdu, Chengdu, China; ^2^ Nursing Department, The People’s Hospital of Wenjiang Chengdu, Chengdu, China; ^3^ The Center of Gerontology and Geriatrics, West China Hospital, Sichuan University, Chengdu, China; ^4^ National Clinical Research Center for Geriatrics, West China Hospital, Sichuan University, Chengdu, China; ^5^ College of Food Science and Engineering, Northwest A&F University, Yangling, China; ^6^ Department of Neuroscience, Baylor College of Medicine, Houston, TX, United States; ^7^ Department of Critical Care Medicine, Sichuan Academy of Medical Science and Sichuan, Chengdu, China; ^8^ Chongqing Key Laboratory of Big Data for Bio Intelligence, Chongqing University of Posts and Telecommunications, Chongqing, China

**Keywords:** elderly, gut microbiota dysbiosis, influencing factors, traditional Chinese medicine intervention, Western medicine intervention, research progress

## Abstract

As the population ages, intestinal health in the elderly has become a key area of concern, with gut microbiota dysbiosis emerging as a significant issue. This review summarizes the factors influencing dysbiosis and interventions from both traditional Chinese medicine (TCM) and Western medicine, offering a reference for future research. A comprehensive search of global databases up to March 2024 identified 617 original studies on gut microbiota dysbiosis in individuals aged 65 and older. After applying strict PRISMA guidelines, 20 articles met the inclusion criteria. Key findings are summarized in four areas: 1) the definition and mechanisms of dysbiosis, 2) evaluation tools for gut microbiota imbalance, 3) factors contributing to dysbiosis in the elderly, and 4) pharmacological treatments. Both TCM and Western medicine offer unique advantages in managing gut microbiota dysbiosis, and the choice of intervention should be tailored to the individual’s condition. Future research should focus on optimizing integrated TCM and Western medicine approaches to improve outcomes for elderly patients with gut microbiota dysbiosis.

## 1 Introduction

Population aging is a global population development trend that brings new challenges to economic and social development (Lauritano et al., 2019). The advent of an aging society significantly increases the incidence of age-related diseases, medical expenses, and social care and security costs, imposing a heavy burden on the country’s economic development. Due to the late development and weak research in geriatrics in China, there is limited evidence, resulting in two-thirds of the elderly population living with diseases, with 150 million suffering from chronic diseases and nearly 40 million disabled. Studies predict that by 2030, health issues in the elderly will offset a 7% increase in China’s annual per capita GDP (Wang et al., 2022). By 2020, direct medical expenses for chronic diseases will rise to 4.2731 trillion yuan, reaching 93.0188 trillion yuan by 2050. Thirty percent of elderly patients consume 70% of medical resources (Zhao et al., 2020). Therefore, with the continuous global aging population, effectively preventing and treating disabilities in the elderly, achieving “ill but not disabled,” and promoting healthy aging have gradually become focal points of medical research and public health (Xu et al., 2022).

The gut microbiota is closely related to human health and longevity (O’Toole and Jeffery, 2015). The mechanism of human longevity is still unclear, but from the perspectives of molecular biology, biochemistry, geriatrics, gerontology, and inorganic nutrition, epidemiological surveys can reveal factors affecting longevity (Ragonnaud and Biragyn, 2021; Ticinesi et al., 2017). The relationship between nutrition and aging is currently a popular topic in unlocking the secrets of human life. Therefore, studying the lifestyle and dietary habits of long-lived populations is essential. With years of research in gut microecology, the significant role of the gut microbiota in organisms has been widely recognized (Pedroza Matute and Iyavoo, 2023). The gut microbiota consists of over 1,000 species and more than 7,000 different strains, with a total number ten times that of human cells, forming a vast and diverse ecological community in the gut (Miner and Kryger, 2017). The gut microbiota is closely related to the occurrence of human diseases and health and longevity. Research on longevity can help us understand how humans delay or overcome the most common age-related diseases, with the gut microbiota gradually becoming a key regulatory factor in various metabolic, immune, and neuroendocrine pathways.

Gut microbiota dysbiosis, a common health issue in the elderly, usually refers to the disruption of the microbial community balance in the intestine, characterized by a decrease in beneficial bacteria and an increase in harmful bacteria. This phenomenon not only directly affects the quality of life of the elderly, leading to issues such as indigestion and nutritional absorption disorders, but is also closely related to the occurrence and development of various chronic diseases (Alhatemi and Savaş, 2023). For example, potential associations exist between gut microbiota dysbiosis and cardiovascular diseases, diabetes, and autoimmune diseases (Liu et al., 2024; Tan et al., 2023). Therefore, in-depth research and discussion on the factors affecting gut microbiota dysbiosis in the elderly and effective intervention measures are of great theoretical and practical value for improving the health level of the elderly, delaying the aging process, and preventing and treating related chronic diseases.

From an academic perspective, researching gut microbiota dysbiosis in the elderly requires considering a variety of factors, including age, dietary habits, lifestyle, chronic disease status, and medication use. At the same time, rigorous scientific methods and in-depth clinical research are needed for the effect assessment and mechanism exploration of different intervention measures (such as drug treatment, diet adjustment, gut microbiota transplantation, etc.) (Islam et al., 2023). In recent years, with the deepening of medical research, people’s understanding of the relationship between the gut microbiota and human health has become more profound. Gut microbiota dysbiosis is closely related to the occurrence and development of various diseases, such as intestinal inflammation, diabetes, cardiovascular diseases, etc (Nesci et al., 2023). Gut microbiota dysbiosis is closely related to the occurrence and development of various diseases, the elderly population, due to the decline in physiological functions and the prevalence of chronic diseases, is more prone to gut microbiota dysbiosis (Ni et al., 2017). In terms of intervention measures, traditional Chinese medicine (TCM) and Western medicine each have their unique advantages. Western medicine mainly improves gut microbiota dysbiosis through drug treatment and diet adjustment, while TCM emphasizes overall conditioning, using a variety of methods such as herbal medicine, acupuncture, and massage to achieve therapeutic purposes. In recent years, with the deepening of integrated traditional Chinese and Western medicine research, the clinical application of combined treatment for gut microbiota dysbiosis has also made significant progress.

Centenarians are the epitome of health and longevity among the elderly. Numerous studies, both domestically and internationally, have reported on the relationship between the gut microbiota of centenarians and health. Dysbiosis of the gut microbiota is associated with major diseases such as obesity, type 2 diabetes, cardiovascular diseases, non-alcoholic fatty liver disease, and cancer. Clea and colleagues conducted a study where they transplanted fecal microbiota into prematurely aging mice to extend their health span and lifespan. The results indicated a connection between aging and the gut microbiota, providing a theoretical basis for microbiota-based interventions in age-related diseases (Li et al., 2021). YOKO and colleagues studied 160 Japanese centenarians and found that specific bile acid metabolism might reduce the risk of pathogenic infections, thereby maintaining gut homeostasis (Johansen et al., 2023). The unique members of the gut microbiota in centenarians not only represent the result of aging but also actively promote resistance to infections or other environmental stresses. Toshitaka and colleagues’ study on the gut microbiota of individuals aged 0-104 showed that the composition of the gut microbiota changes with age, indicating certain patterns and transition points (Odamaki et al., 2016). Gut nutrients might play a significant role in the age-related changes in the gut microbiota composition. In fact, the disruption of host-gut microbiota homeostasis is associated with inflammation, increased gut permeability, and a general decline in skeletal and cognitive health. The gut microbiota is considered one of the variables that can monitor and potentially support healthy aging. However, the exact role of the gut microbiota in aging remains to be fully elucidated.

Research on the factors affecting gut microbiota dysbiosis in the elderly and the progress of traditional Chinese and Western medical interventions is of great significance. This paper aims to summarize and analyze the main factors affecting gut microbiota dysbiosis in the elderly and the research progress of traditional Chinese and Western medicine in intervening gut microbiota dysbiosis, hoping to provide a useful reference for the gut health management and disease prevention of the elderly population.

## 2 Materials and methods

To delve into the impact of gut microbiota disruptions and metabolic status on the health of elderly individuals aged ≥65 years, a detailed search strategy was devised targeting the CNKI, PubMed, Embase, and Medline databases. The following search strings were utilized to retrieve articles published up to March 2024: (“microbiota” or “microorganisms”) AND (“gut” or “feces” or “gastrointestinal tract”) AND (“elderly” or “aging” or “elderly population”). This search strategy ensured comprehensive and targeted retrieval of studies related to gut microbiota disruptions and metabolic status in relation to health outcomes in the elderly population.

### 2.1 Inclusion and exclusion criteria for literature

The inclusion criteria must encompass the following: Research Topic: The literature must directly focus on gut microbiota disruptions in the elderly population (≥65 years). The literature should explore the influencing factors of gut microbiota disruptions in the elderly, including but not limited to age, diet, medication use, chronic diseases, lifestyle, environmental factors, etc. It should also contain research on Chinese and Western medical interventions and advancements for gut microbiota disruptions, such as herbal treatment, acupuncture, massage, and other traditional Chinese medicine (TCM) methods, as well as pharmaceutical therapy, probiotic supplementation, fecal microbiota transplantation (FMT), and other Western medical approaches. Research Methods: The included literature should be original research, including clinical trials, observational studies, experimental studies, case-control studies, cohort studies, systematic reviews, meta-analyses, etc. The literature should have clear research methods, experimental designs, and data analysis processes. The following types of literature will be excluded: those based solely on theoretical discussions, non-systematic reviews or meta-analyses, case reports, news reports, and other non-original research; as well as articles containing duplicate data.

### 2.2 Review process

**Figure 1:**
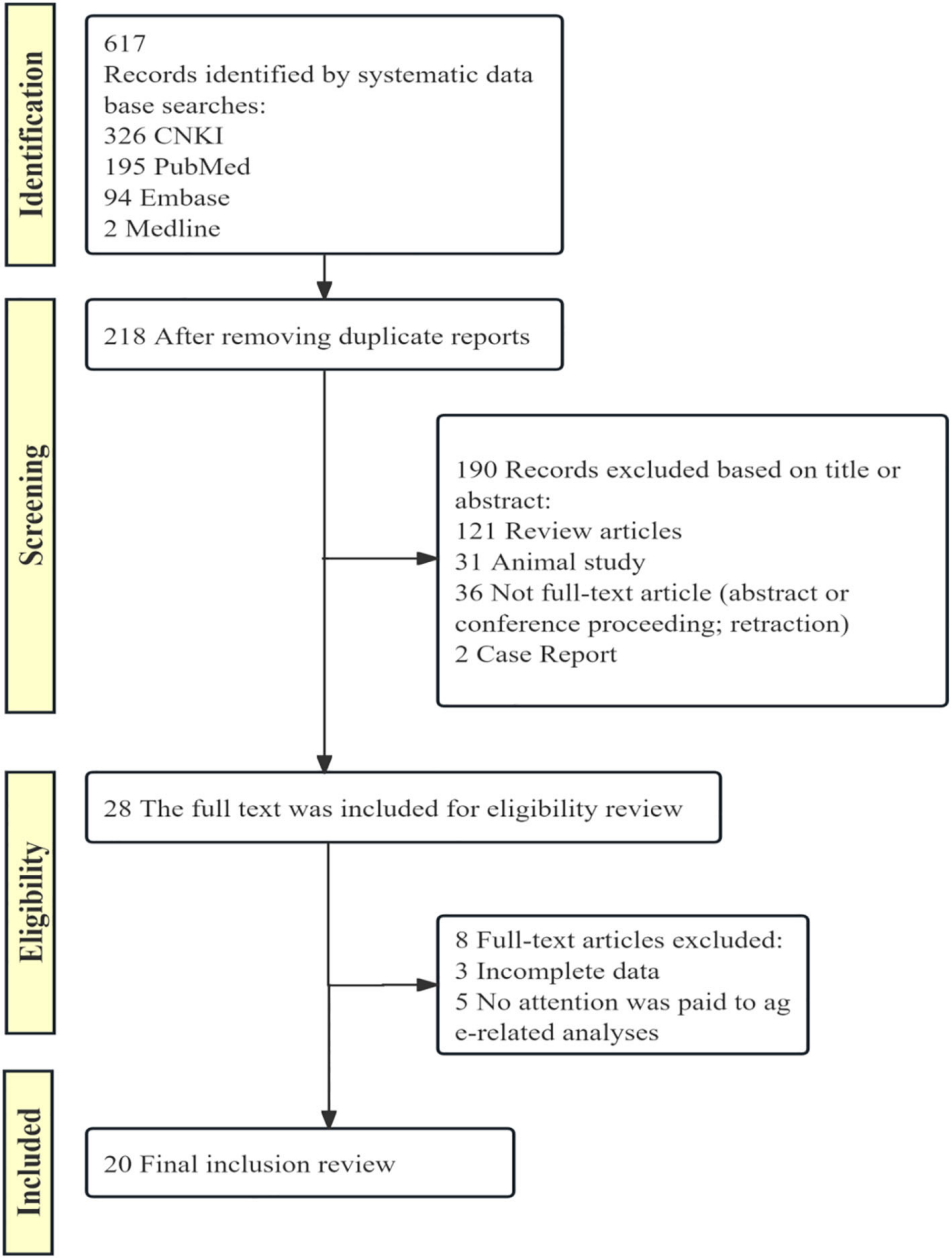
PRISMA flow diagram of study selection process. This flow diagram illustrates the process of identifying, screening, and including studies in the systematic review.

Guided by the rigorous adherence to the PRISMA guidelines, a systematic literature search and screening process was conducted. The initial database search yielded 617 preliminary results. After meticulous initial screening and removal of duplicate reports, a total of 218 relevant articles were identified. Subsequently, based on the preset inclusion and exclusion criteria, a detailed review of the titles and abstracts of the remaining articles was performed. Following this initial screening, 28 articles were selected for in-depth reading and analysis of their full texts. During the full-text eligibility assessment stage, these articles were further evaluated for key elements such as experimental design, data integrity, and validity of the research findings. Ultimately, 20 articles were included as the foundational literature for this study (**Figure 1**).

### 2.3 Search results and description of included studies

The studies are widely distributed, involving multiple countries such as China, the United Kingdom, Singapore, Canada, South Korea, Japan, Finland, Sweden, and the United States. The types of studies include reviews, randomized controlled trials, systematic evaluations, meta-analyses, and others, covering various levels from basic research to clinical applications. The participants range from healthy elderly individuals to those with specific diseases (such as diabetes, dyspepsia, chronic heart failure, etc.), reflecting the complex and intimate relationship between gut microbiota and human health status. Various intervention measures were employed in the studies, including Lycium barbarum polysaccharides, antibiotics (e.g., amoxicillin), probiotics (e.g., Bifidobacterium bifidum, Bifidobacterium longum), prebiotics (e.g., inulin), traditional Chinese medicine prescriptions (e.g., Shenling Baizhu Powder, Guipi Tang, Huanglian Jiedu Tang, Xiangsha Liujunzi Tang), exercise training, and others. These measures were explored to investigate their impact on gut microbiota structure, metabolic activities, and human immune function (**Table 1**).

**Table 1:** Summary characteristics of included systematic reviews.

Basic Information	Country	Study Type and Quantity	Participant Conditions	Interventions	Outcomes
Ying Wang 2021 (Song et al)	China	Review (n=18)	Patients with intestinal diseases such as colon cancer, obesity, type 2 diabetes, autoimmune diseases	Lycium barbarum polysaccharide (LBP)	Regulates immune function by modulating the diversity and structure of the gut microbiota.
Yi Hu 2022 (Hu et al., 2022)	China	Randomized controlled trial (n=119)	Helicobacter pylori-positive dyspepsia or healthy patients	Different doses of amoxicillin and VPZ	H. pylori infection alters the gut microbiota. H-VA treatment has a greater impact on the gut microbiota than L-VA treatment. Gut dysbiosis occurs immediately after eradication but recovers quickly within 4 weeks post-treatment.
S. Macfarlane 2019 (Macfarlane et al., 2013)	UK	Randomized, double-blind, placebo-controlled crossover study (n=43)	Elderly	Probiotics Bifidobacterium longum and inulin-based prebiotic Synergy 1	Short-term use of probiotics effectively improves the composition and metabolic activity of the colonic bacteria and immune parameters in the elderly.
Wei Thwe Khine 2020 (Khine et al., 2020)	Singapore	Randomized controlled trial (n=123)	Community elderly aged 60-85 years	Mindfulness intervention	Elderly patients diagnosed with MCI have specific microbiota distribution profiles. Changes in cognitive function lead to corresponding changes in microbiota distribution, demonstrating the communication direction of the brain-gut microbiota and the feasibility of using gut microbiota as a risk indicator for MCI.
Michelle J. Alfa 2018 (Khine et al., 2020)	Canada	Prospective, placebo-controlled, randomized, double-blind study (n=84)	Community residents (elderly cohort ≥70 years; middle-aged cohort 30-50 years)	MSPrebiotics (unmodified natural RS preparation extracted from potatoes)	MSPrebiotic meets prebiotic standards, increasing the abundance of endogenous bifidobacteria in the elderly and middle-aged without additional probiotic supplementation. Application of MSPrebiotic also eliminates gut dysbiosis observed at baseline in the elderly group.
Chong-Su Kim 2021 (Kim et al., 2021)	Korea	Randomized, double-blind, placebo-controlled multicenter trial (n=63)	Healthy elderly aged ≥65 years	Probiotics containing Bifidobacterium BGN4 and Bifidobacterium longum BOR	Probiotics can enhance mental agility and reduce stress in healthy elderly individuals, while causing changes in the gut microbiota.
Martin C. S. Wong 2023 (Wong et al., 2023)	China	Single-center, double-blind, randomized, placebo-controlled trial (n=497)	Elderly aged ≥65 years or patients with type 2 diabetes	SIM01 treatment	Subjects treated with SIM01 showed a significant increase in beneficial bifidobacteria and butyrate-producing bacteria in stool samples, enhancing the microbial ecological network.
Hirokazu Taniguchi 2018(Hirokazu Taniguchi et al., 2018)	Japan	Randomized crossover trial (n=33)	Elderly males	Endurance training	Short-term endurance exercise has little effect on the gut microbiota of the elderly, with changes in gut microbiota related to cardiometabolic risk factors (e.g., systolic and diastolic blood pressure).
Xiaolin Tong 2018 (Tong et al., 2018)	China	Randomized controlled trial (n=450)	Type 2 diabetes with hyperlipidemia	Metformin and herbal formula	Metformin and the herbal formula improve type 2 diabetes with hyperlipidemia by enriching beneficial bacteria.
Kumail K. Motiani 2019 (Motiani et al., 2019)	Finland	Randomized controlled trial (n=26)	Sedentary subjects (prediabetic, n=9; type 2 diabetes, n=17; aged ≥49 years, n=4; BMI=30.5, n=3)	Exercise training	Gut substrate uptake is related to gut microbiota composition and systemic insulin sensitivity. Exercise training improves gut microbiota and reduces endotoxemia.
Ashley N. Hutchinson 2021 (Hutchinson et al., 2021)	Sweden	Systematicreview (n=4805)	Healthy elderly aged ≥60 years	Probiotics and synthetic microbiota supplements	Probiotics have advantages in altering the gut microbiota composition in healthy elderly individuals and have a moderate effect on immune function.
Liang Luo 2018 (l, 2018)	China	Randomized controlled trial (n=42)	Functional dyspepsia	Shenling Baizhu Powder	Effective in restoring metabolic disorders and gut microbiota dysbiosis in functional dyspepsia.
Fangsheng Ge 2021 (Ge et al., 2021)	China	Randomized controlled trial (n=120)	Elderly patients with type 2 diabetes	Metformin	Regulates gut microbiota imbalance, controls blood sugar and lipid metabolism, and improves inflammatory status by increasing beneficial bacteria.
Yong Zi 2023 (Zi et al., 2023)	China	Randomized controlled trial (n=60)	Elderly patients with chronic heart failure and spleen-heart deficiency	Guipi Decoction	Improves gut microbiota structure and achieves therapeutic effects by modulating gut microecology.
Tong Liu 2023 (Tong et al., 2023)	China	Randomized controlled trial (n=78)	Elderly patients with type 2 diabetes	Huanglian Jiedu Decoction	Improves gut microbiota quantity and insulin-related indicators in patients.
Jinbo Pan 2022 (Jin-bo, 2022)	China	Randomized controlled trial (n=82)	Elderly patients with septic gastrointestinal dysfunction	Xiangsha Liujunzi Decoction	Regulates gut microbiota, improves immune function, reduces inflammatory response, and improves gastrointestinal dysfunction in sepsis.
Shirong Ruan 2023 (Shirong, 2023)	China	Randomized controlled trial (n=50)	Elderly patients with gastric ulcers	Probiotics + anti-Helicobacter pylori	Effective in treating elderly patients with gastric ulcers, promoting Hp clearance and favorably regulating gut microbiota.
Jiaqi Cui 2023 (Qi, 2023)	China	Randomized controlled trial (n=65)	Elderly patients with hypertension	Probiotic Probio-X	Improves gut microbiota structure, increasing Bacteroidaceae and Bacteroides, and decreasing Fusobacteria, Fusobacteria class, α-Proteobacteria class, and unclassified Veillonellaceae.
Larry E. Miller 2018 (Miller et al., 2019)	USA	Systematic review and meta-analysis (n=773)	Healthy elderly individuals	Probiotics	Short-term consumption of probiotics enhances cell-mediated immune function in healthy elderly individuals.
Jelena Vulevic 2015 (Vulevic et al., 2015)	UK	Randomized controlled trial (n=40)	Elderly individuals	Galactooligosaccharide mixture (B-GOS)	Significantly increases Bacteroides and Bifidobacterium, positively impacts immune response, improves NK cell activity, increases anti-inflammatory cytokines IL-10 and IL-8 production, and reduces IL-1β production.

## 3 The concept and mechanism of gut microbiota dysbiosis

### 3.1 The concept of gut microbiota dysbiosis and its interaction with the host

Dysbiosis refers to abnormal changes in the types, quantities, and proportions of the normal gut flora, deviating from the normal physiological combination to a pathological assembly (Bomhof et al., 2010). Clinically, the most apparent symptom of dysbiosis is diarrhea. In addition, it may trigger endogenous infections caused by potential pathogens in the gut flora and a series of allergic diseases, such as atopic dermatitis, allergic dermatitis, and inflammatory bowel disease, which are believed to be related to impaired intestinal barrier function and immune dysregulation caused by changes in the microbiota. The widespread use of antibiotics, especially those with a broad spectrum and long duration of use, has become a major inducer of dysbiosis. Furthermore, treatments such as isotopes, hormones, radiotherapy, and chemotherapy may reduce the body’s immunity and affect probiotics while treating diseases, allowing potential pathogens to colonize (Ramirez et al., 2020). Surgery, trauma, infection, tumors, and environmental degradation may also lead to dysbiosis.

The structure of the gut microbiota, its metabolic products, and its molecular signals are closely related to the occurrence of diseases. With the advancement of metagenomics research, researchers have been able to sequence the DNA of the entire gut microbiota community and analyze these data in detail. Research results show that factors such as aging, dietary changes, and medication use lead to a decrease in the richness and diversity of the gut microbiota, a shift in dominant species, and a decrease in the proportion of probiotics, thereby participating in the formation of various diseases (An et al., 2022). Particularly noteworthy is the significant change in the ratio of Firmicutes to Bacteroidetes, which is considered a significant marker of gut microbiota dysbiosis (Caixia et al., 2010). In the rectum of the elderly, the content of short-chain fatty acids (SCFAs) produced by bacteria that decompose dietary fiber, such as acetate, propionate, butyrate, and valerate, is significantly reduced. These SCFAs play an important role in maintaining the stability of intestinal pH, providing energy for epithelial cells, and maintaining the stability of the gut barrier function (Chonghai, 2007; Hao et al., 2023). Moreover, they exert immunomodulatory effects by activating G-protein-coupled receptors (GPCR43/GPCR41) and the mitogen-activated protein kinase (MAPK) signaling pathway; exert anti-inflammatory effects through the NF-κB pathway; and influence host gene expression by inhibiting histone deacetylases (HDACs). In addition, SCFAs can reduce the recruitment of macrophages and neutrophils by regulating the expression of cytokines in tissues, inhibiting related chemokines, promoting an anti-inflammatory environment in the gut, and enhancing the host’s defense against pathogenic bacteria and pathogens (Jin et al., 2021; Knothe, 1965). Gut microbes can also decompose tryptophan to produce indole-containing metabolites, which regulate the host immune system by activating the aryl hydrocarbon receptor (AHR), promoting anti-inflammatory responses, and maintaining the stability of the host-gut microbiota relationship. These findings not only provide new perspectives for understanding the relationship between gut microbiota and host health but also offer new ideas for future disease prevention and treatment.

### 3.2 Mechanisms behind gut microbiota dysbiosis in the elderly

The gut microbiota in the human body is a complex and delicate ecosystem influenced by many factors, including mode of delivery, daily diet, infection status, and medications (Wei et al., 2021). Over time, the structure and function of the gut microbiota in the elderly undergo significant changes. These changes are reflected not only in the decline in microbial homeostasis and diversity but also in the increase in the total number of facultative anaerobes and the replacement of dominant species. Through culture-dependent and independent studies, scientists have found significant differences in the composition of the gut microbiota between the elderly and younger individuals. These changes do not occur suddenly at a specific age or time point but gradually over the entire lifespan. Compared to healthy elderly individuals, the gut microbiota diversity in frail elderly individuals is even lower, reflecting their declining physical function to some extent (Yang et al., 2010). Through 16S rRNA sequencing, Jafari et al. (2014) found that the number of Bifidobacterium in the elderly decreases with age. Additionally, research by Kato et al. (2016) revealed that during aging, the proportions of Bacteroides, Firmicutes, and Clostridiales increase, while Bifidobacterium is enriched in infants, and Enterobacteriaceae is more common in both infants and the elderly. Notably, with increasing age, the number of butyrate-producing bacteria in the elderly gut significantly decreases, while the number of opportunistic pathogens significantly increases (Cardenas et al., 2014). Butyrate, as a short-chain fatty acid (SCFA), not only serves as an important energy source for colonic cells but also plays a key role in maintaining gut barrier function and exerting anti-inflammatory effects. Therefore, the reduction of butyrate may exacerbate the imbalance of the gut environment in the elderly, increasing health risks (Ding et al., 2021).

Studies have also found differences in gut microbiota types among elderly individuals in different living environments. For example, elderly individuals in the community often exhibit a Prevotella enterotype associated with high fiber intake and low-fat intake, with higher SCFA levels and lower frailty scores (He et al., 2024); while those long-term residents in care institutions often exhibit a Bacteroides enterotype associated with high animal protein and high-fat intake, with reduced gut microbiota diversity, increased levels of inflammatory markers, and lower scores in cognitive and nutritional aspects (Hua et al., 2007). Another study on elderly individuals in Bama, Guangxi, China, showed different results. The study found that centenarians had higher gut microbiota diversity compared to elderly individuals aged 85-89, with increased levels of butyrate-producing Clostridium cluster XIV in the Firmicutes phylum, but decreased levels of Clostridium cluster IV, and an increase in Bacteroides similar to previous studies (Kanauchi et al., 2003). These differences may be attributed to variations in the age range, lifestyle, dietary habits, and detection methods of the study subjects.

The number of microorganisms in the gut far exceeds the total number of human cells, forming a complex biological community that jointly maintains gut health. In this community, beneficial bacteria such as Bifidobacterium and Lactobacillus play crucial roles (Kimoto-Nira et al., 2009). Their metabolic products can regulate signal transmission between immune cells, inhibit the release of pro-inflammatory cytokines, and reduce inflammatory responses; they can also regulate the expression of related oncogenes, inducing apoptosis of cancer cells. The coordination between beneficial bacteria also promotes intestinal peristalsis and mucus flow, effectively resisting the proliferation of harmful bacteria and thus strengthening the gut mucosal barrier function. Conversely, harmful bacteria such as Escherichia coli and Enterococcus may cause inflammation and even cancer (Meng et al., 2023).

Changes in the gut microbiota of the elderly are closely related to their gastrointestinal physiological changes and dietary patterns. With declining immune function, the risk of infection in the elderly also increases accordingly. From infancy to old age, the gut microbiota undergoes a continuous evolution, transitioning from a period of rapid change to a period of regression. During this process, the elderly’s gut responds less to stress, stability decreases, gut microbiota diversity gradually declines, and gut resistance decreases (Pascale et al., 2019). Additionally, the production method of bacterial metabolites changes, shifting from predominantly saccharolytic metabolism to predominantly proteolytic metabolism, leading to a decrease in the levels of short-chain fatty acids in the elderly colon. Besides changes in microbial structure, the gut barrier in the elderly also becomes more permeable. Their intestinal surface area decreases, villi and crypts shorten, intestinal permeability increases, and both the qualitative and quantitative aspects of the mucus layer change (Shuai et al., 2015). These changes are both products of gut microbiota dysbiosis and accelerators of the transport of harmful bacterial products and dysbiosis-related products. Additionally, the reduction in butyrate-producing bacteria in the elderly gut also leads to a decrease in the immune barrier function of the gut mucosa, making it easier for bacteria and their metabolites to enter the bloodstream and cause inflammatory responses. Inflammaging, a state of low-grade, chronic inflammation that occurs naturally during aging, has been shown to be closely related to the development of serious elderly diseases such as cardiovascular diseases, diabetes, and infectious diseases. During this process, the role of the gut microbiota has gradually gained attention from the scientific community. Through a series of gut microbiota transplantation studies in germ-free mice, scientists have found that the aging of the gut microbiota can directly lead to inflammaging. Specifically, when the gut microbiota of young and elderly mice were respectively transplanted into germ-free mice, only the germ-free mice that received the gut microbiota of elderly mice exhibited a phenotype of inflammaging (Shuo et al., 2022; Yonghoon et al., 2023). These findings strongly reveal the causal relationship between gut microbiota and inflammaging. Further research by Kameyama et al. (2001) delved into this mechanism. They found that caloric restriction (CR) could significantly affect the structure of the gut microbiota, thereby reducing the degree of inflammaging, improving the overall health of the host, and extending lifespan. The specific mechanism is that CR increases the abundance of beneficial bacteria such as Lactobacillus and Bifidobacterium, while reducing the abundance of opportunistic pathogens, thus delaying the time for gut antigens to enter the bloodstream and delaying the process of inflammaging, achieving an anti-aging effect.

**Figure 2:**
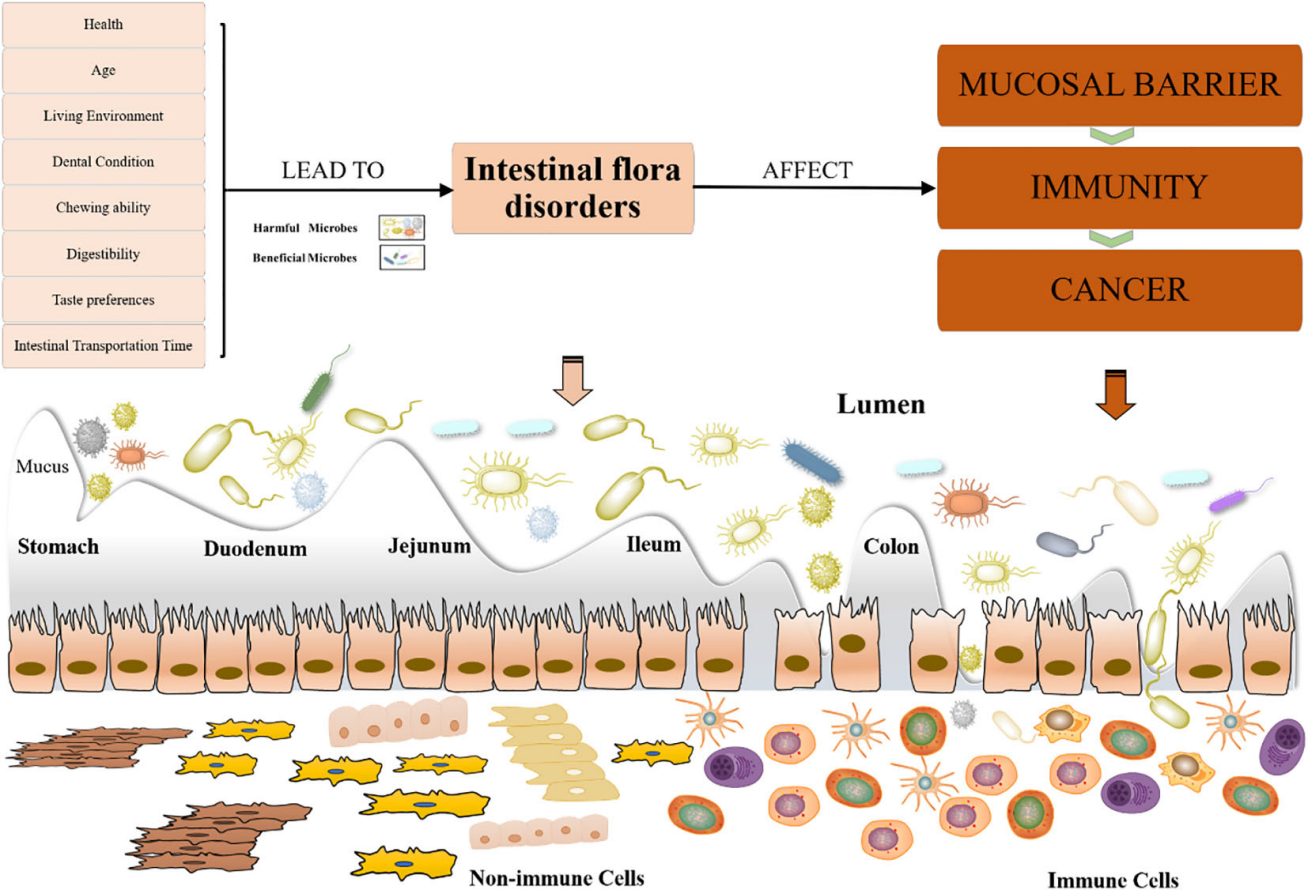
Factors affecting dysbiosis of the gut microbiota in the elderly and its implications. Various factors influence intestinal flora disorders in the elderly and their subsequent effects. Factors such as health, age, living environment, dental condition, chewing ability, digestibility, taste preferences, and intestinal transportation time can lead to dysbiosis of gut microbiota. Dysbiosis, characterized by an imbalance between harmful and beneficial microbes, affects the mucosal barrier, immunity, and increases the risk of cancer. The diagram illustrates the gut sections (stomach, duodenum, jejunum, ileum, colon) and the interaction between microbes and immune cells in the lumen.

Gastrointestinal disorders are a natural physiological change in the aging process of the elderly. With increasing age, the content of pepsin in the stomach and trypsin in the duodenal fluid decreases, the height of the small intestine villi decreases, crypts disappear, and small intestinal peristalsis slows down (Xiujiao et al., 2021). These changes affect the transport, digestion, and absorption of nutrients such as proteins in the gut. In addition, changes in dental condition, chewing ability, taste preferences, digestive ability, and intestinal transit time may affect the food choices and digestion process of the elderly, directly or indirectly leading to changes in the gut microbiota (Zongxin et al., 2023). Therefore, maintaining the balance and health of the gut microbiota is crucial for the elderly (Week et al., 2018). By making reasonable dietary adjustments, increasing physical activity, improving lifestyle, and receiving medical interventions when necessary, the gut microbiota structure of the elderly can be effectively improved, enhancing the quality of life and reducing health risks (**Figure 2**).

### 3.3 Progress in research on the mechanisms of Western and traditional Chinese medicines in regulating gut microbiota

#### 3.3.1 Mechanisms of Western medicines in regulating gut microbiota

In the process of treating diseases, medicines not only act directly on the human body but also exert their therapeutic effects by regulating gut microbiota. In recent years, gut microbiota, as a crucial factor affecting human health and diseases, has received widespread attention. Particularly, hypoglycemic and lipid-lowering drugs and oral probiotics have provided new therapeutic perspectives by regulating gut microbiota and their mechanisms. Recent studies have shown that metformin can also exert hypoglycemic effects by regulating gut microbiota (Shin et al., 2018). It changes the composition and quantity of gut microbiota, increasing the number of bacteria that produce short-chain fatty acids (SCFAs), such as Escherichia coli. These bacteria produce SCFAs that can improve the host’s energy metabolism, thereby indirectly lowering blood glucose (Song et al., 2023). Moreover, metformin can affect the gut microbiota-bile acid-farnesoid X receptor (FXR) axis, reducing the number of Bacteroides and increasing the levels of glycine-ursodeoxycholic acid and tauroursodeoxycholic acid in the gut. These substances act as antagonists of FXR and can improve glucose homeostasis (Sun et al., 2018). Statins, while treating cardiovascular diseases, also exert their therapeutic effects by influencing the structure of gut microbiota. Studies have found that statins can significantly increase the abundance of Bacteroides, Butyrivibrio, and Akkermansia, which are associated with the production of SCFAs that improve the host’s energy metabolism, especially butyrate, which has positive effects on energy metabolism (Chervinets et al., 2022; Huo et al., 2009). Oral probiotics can influence the composition and quantity of gut microbiota, playing a preventive and therapeutic role in metabolic diseases like obesity, mental illnesses, and cancer. Probiotics such as Bacteroides, Lactobacillus, and Bifidobacterium can increase the number of SCFA-producing bacteria in the gut, such as increasing the relative abundance of Bacteroidetes, Lactobacillus, and Bifidobacterium while reducing the relative abundance of Firmicutes. These changes can promote the production of SCFAs, which, by activating G protein-coupled receptors GPR41 and GPR43 on intestinal cells, promote the secretion of peptide YY (PYY) and glucagon-like peptide-1 (GLP-1), inducing satiety, energy expenditure, reducing food intake, and promoting fat reduction (Ilyin et al., 2022; Jiang et al., 2022).

#### 3.3.2 Mechanisms of traditional Chinese medicines in regulating gut microbiota

Traditional Chinese medicines (TCMs) show unique and profound influences in regulating gut microbiota balance and preventing related diseases. As guardians of human health, the balance of gut microbiota is crucial for maintaining various physiological functions of the body. In recent years, with the deepening understanding of the relationship between gut microbiota and human health, the potential of TCMs in regulating gut microbiota has gradually been recognized by the scientific community. TCMs, through their unique active ingredients, can significantly influence the structure, composition, and metabolic products of gut microbiota, thereby playing an important role in preventing and treating various diseases. For instance, in cardiovascular diseases, TCM monomers such as resveratrol, curcumin, and silkworm excrement can inhibit the production of TMAO (a gut microbiota metabolite closely related to cardiovascular disease risk), thereby reducing cardiovascular disease risk, protecting vascular endothelium, and improving lipid metabolism (Runying and Meiqin, 2016; Tellurium et al., 2014). Plantain polysaccharides can increase the abundance of Bacteroides and Bifidobacterium, improving the gut micro-ecological environment and thereby exerting antihypertensive effects (Lu et al., 2024). Additionally, TCMs can effectively inhibit the colonization of pathogenic bacteria and promote intestinal health. Intestinal dysfunction may induce a series of metabolic disorder digestive system diseases, such as celiac disease, colorectal cancer, and inflammatory bowel disease (Chang et al., 2020; Zhuojun et al., 2021). TCMs like Wumei Wan can elevate the B/E ratio of gut microbiota in rats with irritable bowel syndrome models, reduce inflammation factors, and thereby alleviate symptoms of colon mucosal edema and diarrhea (Ding et al., 2019). The sIgA on the intestinal mucosa is the first line of defense of the intestinal mucosa, resistant to various pathogens. TCM components can stimulate the intestinal mucosa to secrete sIgA, enhancing the immune defense capability of the gut and protecting it from pathogen invasion. Meanwhile, TCMs can reduce endotoxin levels and prevent cell apoptosis, which is especially important for patients with chronic kidney disease, alleviating kidney damage and improving prognosis. In terms of glucose and lipid metabolism, TCMs can inhibit α-glucosidase activity, reducing dietary carbohydrate intake, thereby lowering postprandial hyperglycemia and maintaining micronutrient levels in diabetic patients. TCMs like bat excrement and silkworm excrement, regarded as natural regulators of gut microbiota, can improve abnormal glucose metabolism in diabetic animals and prevent chronic complications (Hanbing et al., 2018; Jinli et al., 2020; Xie et al., 2023).

**Figure 3:**
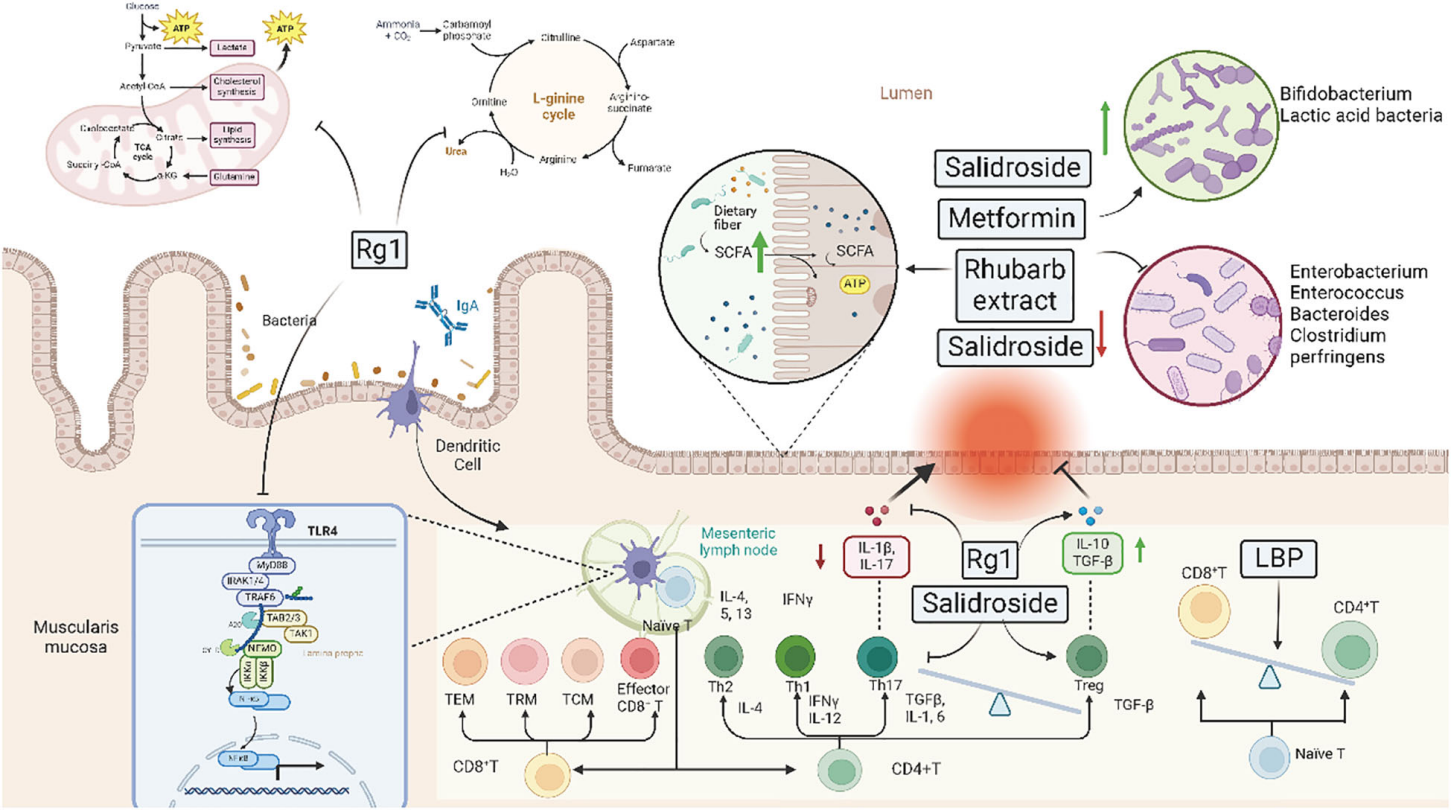
Mechanisms of traditional Chinese medicine and Western medicine in regulating gut microbiota. This figure outlines the mechanisms through which Traditional Chinese Medicine (TCM) and Western Medicine (WM) interventions regulate gut microbiota. The diagram includes metabolic pathways influenced by these interventions, such as the TCA cycle, L-arginine cycle, and SCFA production. Key compounds like Rg1, salidroside, metformin, and rhubarb extract are highlighted for their roles in promoting beneficial bacteria and inhibiting harmful bacteria. The illustration also shows the impact on immune cells, cytokine expression, and gut barrier function, in review demonstrating how these interventions modulate the gut-immune axis to alleviate inflammation and maintain homeostasis.

Clinical aseptic models or fecal transplantation studies further confirm that gut microbiota is a target for TCM treatments. Active ingredients of TCMs can influence the structure, composition, and metabolic products of gut microbiota, thereby achieving the purpose of treating various diseases. However, it is also necessary to note that strong side effects or incorrect combinations of TCMs may exacerbate the condition. Therefore, in the clinical application of TCMs, it is essential to select medicines cautiously and strictly control the dosage and compatibility (**Figure 3**).

## 4 Factors affecting gut microbiota dysbiosis in the elderly

### 4.1 Aging factors

The composition of gut microbiota is not static; it changes dynamically with different life stages (Ottman et al., 2012; Rodríguez et al., 2015). Changes in gut microbiota have significant potential in regulating inflammation and overall health (Belkaid and Hand, 2014; Donald and Finlay, 2023). Gut microbiota dysbiosis is associated with chronic inflammation and age-related syndromes, including cognitive impairment and physical frailty (Cox et al., 2015; Mangiola et al., 2018). It is generally believed that fetuses are sterile during the embryonic stage, as the placenta effectively isolates bacteria and pathogens, keeping the maternal uterus in a sterile environment (Aagaard et al., 2014). After birth, infants acquire their gut microbiota in a complex manner from the external environment (Bäckhed et al., 2015).

The development of gut microbiota in the first 2-3 years after birth can be divided into three periods: developmental (3-14 months), transitional (15-30 months), and stable (≥31 months) (Stewart et al., 2018). These periods are crucial for the maturation and stabilization of gut microbiota. During the developmental phase, the diversity of the gut microbiota in infants undergoes significant changes, predominantly involving five bacterial phyla: Actinobacteria, Firmicutes, Proteobacteria, Bacteroidetes, and Verrucomicrobia (Koenig et al., 2011; Stewart et al., 2018). In the transitional phase, besides changes in microbiota diversity, only Proteobacteria and Bacteroidetes show significant changes (Bokulich et al., 2016). Once reaching the stable phase, the diversity and composition of the gut microbiota in infants stabilize, with few major changes occurring (Lloyd-Price et al., 2017b). During puberty, the gut microbiota also undergoes distinct changes (Vatanen et al., 2016). Animal experiments have shown no differences in gut microbiota composition and diversity between male and female mice before weaning (Markle et al., 2013; Thaiss et al., 2016). However, during sexual maturation, gender differences emerge. Studies have found that while the core gut bacteria in adolescents and adults are the same, the complexity of gut microbiota in adolescents is lower than in adults (Lloyd-Price et al., 2017b; Vatanen et al., 2016). The levels of Clostridium and Bifidobacterium are significantly higher in adolescents, and the functions of gut bacteria differ, with those in adolescents being more related to growth and development, while in adults, they are more associated with inflammation and obesity (Haro et al., 2016; Thaiss et al., 2016).

Adulthood represents a relatively stable period for gut microbiota. The characteristics of adult gut microbiota include a large number, complex composition, but relatively stable structure (Vatanen et al., 2016). The predominant bacteria include Firmicutes, Bacteroidetes, Proteobacteria, and Actinobacteria (Lloyd-Price et al., 2017a). Factors such as stress, antibiotics, and diet can cause changes in the gut microbiota composition in adults, but it can usually recover in a short time to maintain normal gut function (Thursby and Juge, 2017). This state persists into old age (Monda et al., 2017).

With advancing age, the stability and diversity of the gut microbiota diminish, impairing intestinal barrier functions and predisposing hosts to age-related diseases and unhealthy aging processes. Compared to other age groups, the elderly have reduced quantities of lactic acid bacteria, Clostridiales, and Akkermansia, which produce anti-inflammatory short-chain fatty acids (SCFAs) and saccharolytic bacteria, while proteolytic bacteria numbers increase. These changes signify a reduction in the Firmicutes to Bacteroidetes ratio and an increase in subdominant species during aging, leading to a decline in immune system function (immunosenescence) and low-grade chronic inflammation (inflammaging) (Deng et al., 2023). Remarkably, centenarians exhibit a unique gut microbiota profile distinct from typical elderly populations, combining youthful characteristics dominated by Bacteroides with those of the elderly dominated by Escherichia-Shigella. Notably, these centenarians show a balanced gut microbiota, enriched with potentially beneficial species and fewer pathogens. The enrichment of Coprococcus and bacteria from the Odoribacteraceae family in their intestines, efficient producers of the anti-inflammatory bile acid metabolite isoallolithocholic acid, significantly inhibits the proliferation of Gram-positive pathogens, contributing to longevity (Strasser et al., 2021). Additionally, beneficial bacteria like Actinobacteria and Faecalibacterium are significantly enriched in centenarians, showing increased microbial diversity and formidable resistance against aging-related diseases and chronic inflammation.

Given the close link between gut microbiota and aging, modulating the gut flora to decelerate aging processes has become a viable strategy. For instance, the intake of probiotics such as Bifidobacterium and Lactobacillus can effectively improve gut homeostasis, alleviate aging-related inflammatory responses, and thus prevent diseases like hypertension, hyperlipidemia, and diabetes. Furthermore, interventions like oral probiotics, caloric restriction, and fecal microbiota transplantation can regulate the gut microbiota, alleviating age-related inflammation and immunosenescence to promote healthy longevity.

### 4.2 Physical activity factors

In-depth studies on subjects with varying levels of physical activity or fitness have revealed significant differences in gut microbial composition, suggesting that physical exercise may positively influence gut microbiota (Margolis et al., 2021). Research on professional athletes further indicates a significant correlation between high levels of physical exercise and increased α-diversity of the gut microbiome compared to controls (Sampson et al., 2016). Peak oxygen uptake, an important measure of cardiorespiratory health and physical condition, is positively associated with higher α-diversity in healthy, young individuals. However, no significant differences in microbial diversity were found when comparing physically active to sedentary women (Katsirma et al., 2021). A randomized crossover trial in the elderly showed a positive correlation between peak oxygen uptake and α-diversity indices, yet no significant differences in α-diversity were observed between the exercise and control periods (Taniguchi et al., 2018).

In exploring innate immune system cells, cross-sectional studies (Pickard et al., 2017; Wastyk et al., 2021) compared elderly groups with differing levels of physical activity. Regular exercise was found to be associated with enhanced natural killer (NK) cell and neutrophil function, such as increased cytotoxicity and improved neutrophil migration towards IL-8. Controlled intervention studies also demonstrated that exercise influences the characteristics of the innate immune system. For example, improvements in bacterial phagocytic function and neutrophil oxidative burst capacity were observed after 10 weeks of high-intensity interval training (Nagata et al., 2023). Similarly, a 12-week moderate-intensity combined strength and endurance training reduced the proportion of CD14+/CD16+ monocytes, indicating a decrease in pro-inflammatory and aging monocyte subtypes (Shi et al., 2017). Furthermore, exercise has been shown to directly affect the M1 to M2 macrophage transition, down-regulating the inflammatory milieu and promoting anti-inflammatory cytokine secretion (Beam et al., 2021). In summary, an increase in habitual physical activity has a positive regulatory effect on innate immune function, which may help reduce the risk of infections and systemic inflammation.

### 4.3 Dietary factors

Among the many influencing factors, diet is one of the key elements that ensures the orderly metabolism of gut microorganisms, determines the composition and changes of gut microbiota, and is closely related to the occurrence of various chronic metabolic diseases (Makki et al., 2018). Research by Zhang et al. (2010) showed that experimental mice fed different diets had differences in gut bacterial composition. Previous studies have shown that the levels of Klebsiella, Enterococcus, Lactobacillus, and Citrobacter are generally similar among people with different diets. However, pure vegetarians have significantly lower levels of Enterobacteriaceae, Bacteroides, Bifidobacterium, and Escherichia coli compared to those with other dietary patterns (Yu et al., 2023). Additionally, David et al. (2014) found that switching to a pure vegetarian or pure carnivorous diet could alter the composition of the gut microbiota in a short time. A meat-based diet increases the abundance of bile-tolerant microorganisms such as Bilophila and Bacteroides while decreasing the bacteria that metabolize plant polysaccharides, like those in the Firmicutes phylum.

With the rapid economic development and improvement in living standards, changes in dietary structure have become more significant compared to natural environmental factors. High-fat, high-sugar, and high-salt diets have increased, while the intake of dietary fibers from grains has decreased. Unhealthy dietary patterns lead to various adverse outcomes, with high-fat diets being the most studied and widely harmful. Dietary fats, the third largest energy source for humans, affect the structure and metabolic activity of gut microbiota. Imbalances in microbial communities can adversely affect fat digestion and absorption, creating a vicious cycle. Ingested fats combine with bile acids (Collins et al., 2023), converting them into bioactive metabolites absorbable by intestinal epithelial cells. Primary bile acids that escape intestinal absorption undergo microbial-mediated enzymatic hydrolysis in the terminal ileum or colon, producing secondary bile acids. These bile acids are carcinogenic and are associated with the etiology of colorectal cancer and other gastrointestinal diseases. The main microbial groups that dissociate primary bile acids bound to taurine and glycine include Bifidobacterium, Lactobacillus, and Bacteroides (Cai et al., 2022; Cheng et al., 2022; de Vos et al., 2022). Bacteria also produce a significant amount of bioactive lipids, including lipopolysaccharides (LPS), which are components of the cell walls of Gram-negative bacteria. LPS can cause inflammation in the gut and other organs, especially chronic low-grade systemic inflammation, leading to various metabolic diseases (Schoeler and Caesar, 2019).

Research has shown that long-term consumption of animal-based foods increases the abundance of Bacteroides and reduces the abundance of Firmicutes, suggesting that Bacteroides are closely related to protein and fat metabolism, while Firmicutes are mainly associated with the metabolism of polysaccharides and oligomers (Delannoy-Bruno et al., 2021). High-fat diets are often accompanied by high-sugar diets. Studies have found that high-fat, high-sugar diets are closely related to the occurrence of Crohn’s disease. Agus et al. (2018) speculated that Crohn’s disease is associated with an increase in the abundance of mucosa-associated pro-inflammatory Proteobacteria (mainly E. coli) and a decrease in SCFA-producing beneficial bacteria caused by high-fat, high-sugar diets. They also found that the receptor for SCFA, GPR43, is downregulated in mice on high-fat, high-sugar diets and in Crohn’s disease. Transplanting feces from high-fat, high-sugar diet mice into germ-free mice increased susceptibility to E. coli infection. Long-term consumption of high-sugar carbonated beverages may promote autoimmune diseases (such as encephalitis and rheumatoid arthritis), mainly by resident gut bacteria. The segmented filamentous bacteria antigen is recognized and presented by dendritic cells, activating the differentiation of Th17 cells in the mucosal lamina propria, which is an important pathogenesis mechanism of autoimmune encephalitis. 16S rRNA sequencing results showed that in autoimmune encephalitis model mice, the abundance of Mucispirillum schaedleri, Butyricicoccus pullicaecorum, Desulfovibrio C21-C20, and Ruminococcus gnavus increased after consuming high-sugar carbonated beverages, and this effect could be inhibited by caffeine, which mainly suppresses the infiltration of inflammatory lymphocytes. Liu et al. (2018) found that after 15 days on a high-protein diet, the concentration of SCFAs in the colon increased, which was associated with decreased concentrations of Clostridium coccoides, Clostridium leptum groups, and Faecalibacterium prausnitzii. Zhuang Guo et al. (2016) found significant differences in the structure and function of gut microbiota between gout patients and healthy individuals, with higher abundances of Bacteroides caccae and Bacteroides xylanisolvens in gout patients, and significantly lower abundances of Faecalibacterium prausnitzii and Bifidobacterium, suggesting that gut microbiota analysis could aid in the early diagnosis of gout. Li et al. (2012) found that high-salt diets increase the Firmicutes/Bacteroidetes ratio, increase the abundance of Lachnospiraceae and Ruminococcus, and decrease the abundance of Lactobacillus, affecting protein digestion. High-salt diets also induce and exacerbate ulcerative colitis and neuroinflammation by changing gut microbiota, activating Th17 immune cells in the intestinal mucosa and promoting IL-17 secretion, similar to the mechanism by which high-sugar diets cause autoimmune diseases. High dietary fiber intake is the preferred dietary pattern for weight loss and maintaining gut health. Its effects, similar to the addition of prebiotics, mainly promote the growth of beneficial bacteria and increase SCFA concentration in the intestinal cavity, preventing metabolic syndrome caused by high-fat diets. High-protein diets do not have this effect.

With age, individuals suffering from malnutrition often experience severe gut microbiota destruction, leading to gradual wasting through mechanisms like synthetic metabolic resistance, malabsorption, anorexia induction, and reduced vitamin synthesis (Kinross and Nicholson, 2012). A middle to high protein intake of 1-1.2 g/kg body weight per day is considered key to the nutritional needs of the elderly, aiding in protein synthesis and preventing frailty. Yet, from the gut microbiota’s perspective, studies show that increasing protein intake, particularly animal proteins, can cause dysbiosis, reducing SCFA-producing bacterial populations and raising levels of trimethylamine N-oxide (TMAO), a marker for elevated cardiovascular risk (Magrone and Jirillo, 2013). A middle to high protein intake of 1-1.2 g/kg body weight per day is considered key to the nutritional needs of the elderly, aiding in protein synthesis and preventing frailty. Yet, from the gut microbiota’s perspective, studies show that increasing protein intake, particularly animal proteins, can cause dysbiosis, reducing SCFA-producing bacterial populations and raising levels of trimethylamine N-oxide (TMAO), a marker for elevated cardiovascular risk (Makki et al., 2018). It’s worth noting, however, that the gut microbiome is affected not only by the amount of dietary protein but also by its quality, timing of consumption, nitrogen availability, and the levels of other nutrients like carbohydrates and fiber. Studies in the elderly and sedentary adults show that increased protein intake is not associated with significant adverse changes in gut microbiota composition in real-life settings, but extremely high protein levels can significantly elevate the cardiovascular risk marker TMAO in elderly participants (Berding et al., 2021). Hence, for healthy aging, protein intake should be kept within 1-1.2 g/kg body weight per day, focusing on proteins of high biological value, coupled with sufficient intake of carbohydrates and fiber. Fiber intake helps to promote gut microbiota diversity and the growth of SCFA-producing species like Bifidobacterium. SCFAs have various metabolic functions in the body, including regulating synthetic metabolism, improving insulin sensitivity, and providing anti-inflammatory effects. Considering that synthetic metabolic resistance, oxidative stress, and inflammation are closely associated with aging, ample synthesis of SCFAs by the gut microbiome may help mitigate the adverse effects of these pathways (Fan et al., 2023). Fruits and vegetables, as primary sources of fiber and polyphenols, contribute to regulating oxidative stress, improving intestinal permeability, and balancing the gut microbiota once metabolized by gut microbes. Exploring the diet of populations in longevity regions, Bama Yao Autonomous County in Guangxi, China, is known as a longevity region with a high proportion of centenarians. The local residents’ diet can be roughly divided into three categories: porridge, soup, and fruits and vegetables. They have long consumed locally grown crops such as corn, red sweet potatoes, hemp seeds, and pumpkins. Their staple food mainly consists of porridge mixed with corn, sweet potatoes, and pumpkins. Corn from Bama, Guangxi, is known as “yellow pearl corn” due to its rich nutritional content, higher fat and protein content compared to regular wheat and rice, high dietary fiber content, low calorie count, and its ability to increase intestinal peristalsis, reduce constipation, and minimize toxin absorption (Deng et al., 2019). The primary daily drink in Bama is hemp seed soup, referred to locally as “longevity soup,” which is a favored delicacy among the ethnic minorities in Bama (Li et al., 2022a. Hemp seeds belong to the Moraceae family and are a valuable oil crop grown in the mountainous areas of Bama. Hemp seeds have various medicinal benefits, such as treating constipation in the elderly and lowering serum cholesterol, blood lipids, and blood pressure. Vegetables and fruits constitute a significant part of the diet in Bama’s longevity region. Common and favored vegetables include pumpkin, pumpkin leaves, sweet potato leaves, green vegetable leaves, bitter hemp, and wild vine vegetables. The fruits they frequently consume include guava, mountain peaches, bananas, loquats, and citrus. In summary, the traditional diet in Bama’s longevity region mainly consists of plant-based foods such as coarse grains, vegetables, and fruits, resulting in a high intake of dietary fiber. Their gut microbiota also contains a high number of beneficial bacteria, such as Lactobacillus, and the diversity of probiotics like Bifidobacteria and lactic acid bacteria is more abundant (Li et al., 2022b). The Mediterranean diet, combining a balanced intake of high biological value proteins, complex carbohydrates, fiber, and polyphenols, is an ideal dietary pattern. Adherence to the Mediterranean diet has been linked to increased gut microbiota diversity, improved symbiont-pathogen balance, and elevated SCFA production (Ristori et al., 2019).

## 5 Pharmacological treatment of gut microbiota dysbiosis in the elderly

### 5.1 Traditional Chinese medicine

#### 5.1.1 TCM formulas

Zi et al. (Z et al., 2023) investigated the effects of Gui Pi Tang on the cardiac function and intestinal microbiota of elderly patients with chronic heart failure and spleen deficiency. The results showed a decrease in the relative abundance of Bacteroides, Shigella, Escherichia coli, Clostridium perfringens, Lactobacillus, and Veillonella in the treatment group, while the relative abundance of Prevotella, Bifidobacterium, Lactobacillus, and Parabacteroides increased after treatment (*P* < 0.05). Liu et al. (2023) studied the therapeutic efficacy of Huanglian Jiedu Tang plus-minus in elderly patients with type 2 diabetes mellitus (T2DM) and its effects on patients’ glucose and lipid metabolism and intestinal microbiota. It was found that after 2 months of treatment, the quantities of Lactobacillus, Bifidobacterium, and Lactobacillus increased in both groups, while the quantities of yeast, Enterobacteriaceae, and Enterococcus decreased. Pan (Jinbo, 2022) demonstrated that in elderly septic patients receiving nasal feeding of Xiang Sha Liu Jun Zi Tang in addition to the control group, the increment of Lactobacillus and Bifidobacterium in the Chinese medicine group was higher than that in the control group, while the decrement of Enterobacteriaceae, Enterococcus, Bacteroides, and Clostridium difficile was higher than that in the control group (*P* < 0.05).

Clinical sterile models and fecal transplantation therapy have proven that the intestinal microbiota can be targeted by traditional Chinese medicine to achieve the goal of disease treatment, mainly manifested in the influence of effective components of Chinese medicine on the structure, composition, and metabolites of the intestinal microbiota. Specifically, this involves increasing the abundance of beneficial bacteria such as Bifidobacterium and Lactobacillus, while reducing the abundance of harmful bacteria such as Bacteroides and Vibrio species. Chinese medicine can inhibit the production of lipopolysaccharides (LPS) by the intestinal microbiota, increase the levels of short-chain fatty acids (SCFAs) in the intestine. SCFAs are considered hallmark substances of intestinal barrier damage, and increasing their levels can restore the damaged intestinal barrier system. This can inhibit the passage of bacteria and their metabolites through the damaged intestinal barrier to the liver, thereby exacerbating liver inflammation. The aim is to protect and alleviate liver damage (Schneider et al., 2000).

#### 5.1.2 Single herbs

The aqueous extract of Phyllanthus emblica L. (APE) can enhance the relative abundance of beneficial gut bacteria like Lactobacillus and Bifidobacterium, while reducing the abundance of harmful bacteria such as Fusobacteria and another branch of the bacteria, restoring homeostasis and suggesting an ameliorative effect on functional dyspepsia (FD) (Xp, 2022). Zheng’s thorough research indicates that Liushenqu significantly alters the gut microbial composition at the phylum level. Rich in digestive enzymes, yeasts, other beneficial microbes, and a variety of miscellaneous bacteria, this TCM ingredient effectively promotes the expression of beneficial microbes, thus aiding the recovery of gut microbiota imbalance to a normal state. Compared to the FD group, rats treated with 3g/kg Liushenqu showed a significant increase in the ratio of Firmicutes to Bacteroidetes. Additionally, Liushenqu inhibited the production of total SCFAs as well as acetic acid, isobutyric acid, butyric acid, and caproic acid in the rat colon. Based on these findings, the authors hypothesized that Liushenqu might exert its therapeutic effect on FD symptoms by modulating the gut microbial structure and subsequent SCFA changes in FD rats, thus affecting immune functions. Wang et al. (W et al., 2021) used cyclophosphamide-induced immunosuppressed mice and RAW 264.7 cells to investigate the immunomodulatory effects of Lycium barbarum polysaccharide (LBP) both *in vivo* and *in vitro*, along with LBP’s transmembrane transport in the gut, to explore the immune activity and pathways of LBP’s immunomodulatory action. As polysaccharide structure is closely linked to activity, establishing quality control methods reflecting structure and purity is vital for the effectiveness, stability, homogeneity, and safety of polysaccharide products. Zi et al. (2023) found through 16S rRNA gene sequencing that salidroside can restore the diversity of gut microbiota in UC mice, increase the proportion of bacteria such as Firmicutes, suppress Bacteroidetes, significantly increase the number of Treg cells in the colonic lamina propria, and reduce the levels of IL-6 and IL-17A mRNA expression, inhibiting Th17 differentiation, leading to reduced production of the pro-inflammatory cytokine IL-1β to alleviate inflammatory damage to the intestinal mucosa. Ginsenoside Rg1, an extract from ginseng, has been found by Zhang et al. to regulate various metabolic pathways in the gut microbiota (such as inhibiting the glyoxylate cycle, citric acid cycle, and L-arginine pathway), suppress Th17 expression, promote Treg differentiation, down-regulate the Toll-like receptor 4/myeloid differentiation factor 88 signaling pathway, thus lowering serum levels of pro-inflammatory cytokines IL-1β, TNF-α, and IL-17A, and increasing the anti-inflammatory cytokine IL-10 level, thereby repairing inflammatory damage to the intestinal wall. Aqueous extracts of Phyllanthus emblica L., Liushenqu, LBP, salidroside, and ginsenoside, along with other TCM ingredients like baicalin, matricin, and rhein, have all shown positive modulation of gut microecology and the immune system. Moreover, they have been demonstrated to improve the diversity and distribution of gut microbiota, offering new perspectives and strategies for the application of TCM in the treatment of related diseases (in **Table 2**).

**Table 2:** Progress in clinical application of traditional Chinese medicine interventions for gut microbiota dysbiosis in the elderly.

No.	Medication/Formula	Composition	Sample Size	Intervention	Main Results
1	Jianpi Jiangzhuo Formula	Poria 21 g, Codonopsis 18 g, Cinnamon Twig 15 g, Atractylodes Macrocephala 15 g, Atractylodes 15 g, Hawthorn 15 g, Tangerine Peel 9 g, Pinellia 12 g, Red Yeast Rice 9 g, Salvia 9 g	32 male rats	The Jianpi Jiangzhuo group was administered 4.0g/100g body weight once daily by gavage (equivalent to 10.8 times the clinical adult dose); the ranitidine group was administered 2.7mg/100g body weight once daily by gavage	Compared with the normal control group, the EGF and PGE levels in the blood of the model group were decreased (P<0.05)
			127 elderly patients with metabolic syndrome (MS)	The control group received conventional symptomatic treatment with Western medicine, while the observation group received the Jianpi Jiangzhuo Formula in addition to the control group treatment	Poria can strengthen the spleen and promote diuresis, Salvia can invigorate blood circulation, and Tangerine Peel can resolve dampness and phlegm. It improves gut microbiota imbalance by regulating the gut microbiota composition and repairing small intestine villi.
2	Guipi Decoction	Astragalus 15 g, Codonopsis 15 g, Atractylodes Macrocephala 15 g, Angelica 12 g, Poria 15 g, Raw Rehmannia 15 g, Cinnamon Twig 10 g, Aucklandia 9 g, Ziziphus 10 g, Honey-fried Licorice 12 g	56 female rats	The model group, fluoxetine group, and low, medium, and high dose Guipi Decoction groups were administered respective interventions by gavage for 3 weeks	Guipi Decoction can increase body weight and improve depression-like behavior in rats, potentially by modulating gut microbiota structure and composition.
			60 elderly patients with chronic heart failure (CHF)	Both groups received conventional Western medicine treatment, and the treatment group additionally received a modified Guipi Decoction	The treatment group had a lower abundance of Bacteroides (P<0.05) and higher abundances of Blautia, Prevotella, and Roseburia (P<0.05) compared to pre-treatment.
3	Huanglian Jiedu Decoction	Coptis 9 g, Scutellaria 6 g, Gardenia 9 g, Phellodendron 6 g	90 SPF SD rats	Divided into high, medium, and low dose Huanglian Jiedu Decoction groups, blank control group, and antibiotic group. Each group had 18 rats. The medium dose was based on the adult dose (0.5 g/kg). The high dose was twice the medium dose, and the low dose was half the medium dose.	The high dose Huanglian Jiedu Decoction group had fewer E. coli than the normal group.
			40 elderly patients with damp-heat type anal fistula	Both groups received conventional Western medicine treatment, and the traditional Chinese medicine group additionally received Huanglian Jiedu Decoction orally for 2 weeks	Huanglian Jiedu Decoction effectively increased Actinobacteria and Cyanobacteria and inhibited Fusobacteria, providing systemic and local anti-inflammatory effects for anal fistula.
4	Berberine Hydrochloride	-	80 SPF male mice	The TAB group was given 100 mg·kg-1 by gavage; H-SPI group and L-SPI group were given 100 mg·kg-1 and 50 mg·kg-1 by gavage; CG group and MG group were given 0.5% CMC-Na solution of equal volume by gavage.	Compared to the MG group, the TAB, H-SPI, and L-SPI groups had significantly increased relative abundances of Akkermansia and Bacteroides (P<0.05) and significantly decreased relative abundance of E. coli (P<0.05).
			96 elderly patients with T2DM	The control group received metformin in addition to conventional treatment, while the observation group received berberine hydrochloride in addition to the control group treatment for 3 months	Post-treatment, the observation group had higher levels of Lactobacillus and Bifidobacterium, and lower levels of Enterococcus and Enterobacteriaceae compared to the control group (P<0.05).
5	Rhubarb	-	50 SPF male SD rats	The rhubarb anthraquinone group received 100 mg/kg rhubarb anthraquinone by intraperitoneal injection. [11]。The metformin group received 200 mg/kg metformin by intraperitoneal injection. The rhubarb anthraquinone + metformin group received 100 mg/kg rhubarb anthraquinone and 200 mg/kg metformin by intraperitoneal injection daily for 2 weeks.	Compared to the model group, the relative abundances of Lactobacillus and Bifidobacterium were significantly increased in the rhubarb anthraquinone group (q=11.555, 17.876, both P<0.001). Compared to the rhubarb anthraquinone group, the relative abundances of Lactobacillus and Bifidobacterium were significantly decreased in the rhubarb anthraquinone + metformin group (q=9.941, 10.809, both P<0.001), and the relative abundance of E. coli was significantly increased (q=8.874, P<0.001).
6	Aged Fuzhuan Tea Polyphenols	-	Gut microbiota from 3 elderly individuals (65 years old) in mixed culture medium	Add 2.50 g (purity 83.15%) of 1-year aged Fuzhuan tea polyphenols and 2.73 g (purity 76.56%) of 7-year aged Fuzhuan tea polyphenols to mixed culture medium, vortex for 3 min, and mix thoroughly to obtain 1-year aged tea polyphenols group (N group) and 7-year aged tea polyphenols group (O group)	The O group significantly increased the contents of acetate, propionate, and butyrate, as well as the abundance and diversity of the elderly gut microbiota compared to the N group and B group.
7	Rhodiola	-	60 male rats	Preventive intraperitoneal injection of salidroside, salidroside + dorsomorphin (50 mg/kg, 20 mg/kg, 50 + 20 mg/kg), followed by therapeutic administration for one week	Salidroside reshapes the gut microbiota by regulating endogenous bacteria (e.g., Ruminococcaceae, Oscillospira, Lachnospiraceae, and Akkermansia) and improves gut microbiota dysbiosis.
8	Ginseng	-	90 SPF female mice	Except for the two normal groups, the remaining groups of mice were subcutaneously injected with D-galactose (140 mg/kg) combined with intragastric administration of AlCl3 (20 mg/kg); the ginseng protein group received the corresponding solution at 0.1 g/kg by gavage, once daily for 30 days	Ginseng protein affects bacterial abundance, making the gut microbiota closer to normal levels and reducing the abundance of Alzheimers disease-related bacteria such as Alloprevotella, Ruminococcus_1, and Prevotellaceae_UCG-001.

### 5.2 Western medicine

#### 5.2.1 Probiotics

Probiotics are beneficial microorganisms that adhere to the epithelial cells of the host’s intestinal mucosa. They maintain the stability of the intestinal microecology while resisting pathogenic bacteria. Additionally, probiotics secrete a diverse array of digestive enzymes that facilitate the breakdown of complex food components that would otherwise be difficult for the body to process. For example, strains such as Lactobacillus assist in lactose digestion, which is particularly advantageous for individuals with lactose intolerance. This can alleviate discomforts including diarrhea and bloating that result from lactose malabsorption. Research indicates that probiotics can reverse intestinal permeability by increasing the production of tight junction proteins (Rampelli et al., 2020). The most commonly used bacterial genera of probiotics are Lactobacillus and Bifidobacterium. Probiotics effectively reduce the proliferation of Helicobacter pylori (HP) in the stomach and form a protective film on the gastrointestinal mucosa, thereby reducing the occurrence of infections. Ruan et al. (Shi-rong, 2023) conducted a study in which probiotic therapy was added to the treatment regimen for Helicobacter pylori in the observation group compared to the control group. Clinical efficacy, HP eradication rate, gastric function before and after treatment, and the status of intestinal microbiota were compared between the two groups. The results showed that the total effective rate in the observation group was 96.0%, and the HP eradication rate was 94.0%, both higher than those in the control group (82.0% and 80.0% respectively), indicating that probiotics have a desirable regulatory effect on patients’ gastric function and intestinal microbiota. Probio-X compound probiotics include Lactobacillus casei Zhang, Bifidobacterium animalis V9, Lactobacillus plantarum P-8, Bifidobacterium animalis M8, and Lactobacillus rhamnosus M9. Through a three-month randomized controlled trial (RCT), Cui (Qi, 2023) evaluated the adjunctive treatment effects of Probio-X on elderly hypertensive patients and its impact on their intestinal microbiota. The results showed that Probio-X probiotics could improve the structure of intestinal microbiota in elderly hypertensive patients, with an increase in the family and genus of Bacteroidaceae and a decrease in the phylum, class, order of Clostridiaceae, and unclassified genus of Bifidobacteriaceae.

#### 5.2.2 Metformin

Metformin belongs to commonly used antidiabetic drugs, which not only inhibits hepatic gluconeogenesis but also enhances peripheral tissue insulin sensitivity. It possesses certain anti-tumor and anti-inflammatory effects and influences the composition of intestinal microbiota, thereby improving glucose metabolism and energy balance. Ge et al. (2021) found that after treatment with metformin, the quantities of Bifidobacterium, Lactobacillus, and Bacteroides in the intestine significantly increased compared to before treatment, while the quantities of Enterococcus, Enterobacteriaceae, and yeast significantly decreased. It is evident that metformin can markedly increase the number of probiotics, reduce harmful bacteria, and ameliorate the imbalance of intestinal microbiota. Metformin promotes the proliferation of certain specific bacteria (such as Lactobacillus) while inhibiting the growth of others (such as Enterobacteriaceae), effectively adjusting the composition of the intestinal microbial community. Furthermore, *in vitro* studies (Ruth, 2014) have demonstrated that metformin has a direct and significant impact on the growth of adherent Bifidobacterium and adolescent Bifidobacterium. In the intestinal milieu, metformin not only enhances the efficiency of glucose uptake but also promotes the generation of short-chain fatty acids (SCFAs), thereby reinforcing intestinal barrier function and finely regulating intestinal peptide secretion. These combined effects highlight the significant role of metformin in maintaining intestinal health.

#### 5.2.3 Enteral nutrition

Enteral nutrition (EN) is a method of providing necessary nutrients and other essential substances for metabolism through the gastrointestinal tract. Peng et al. (Aiming et al., 2023) explored the effects of full-protein enteral nutrition based on Nutritional Risk Screening (NRS) on the nutritional status, intestinal microbiota, and peripheral blood balance of helper T cells 17 (Th17)/regulatory T cells (Treg) in elderly patients with liver cirrhosis. The results showed that after 3 months of treatment, the serum total protein (TP), albumin (ALB), and prealbumin (PA) levels increased in both groups, with higher levels in the study group than in the control group (P < 0.05). Full-protein enteral nutrition, as an efficient nutritional support method, has multiple positive effects. It not only stimulates the secretion of digestive juices and gastrointestinal hormones to maintain the normal growth of indigenous gut flora but also effectively promotes gastrointestinal motility, further improving and maintaining the integrity of intestinal mucosal cell structure and function (Santarpia et al., 2007). These physiological effects demonstrate the significant advantage of full-protein enteral nutrition in regulating intestinal microbiota. Additionally, full-protein enteral nutrition can provide comprehensive and rich nutrients to the body, effectively preventing bacterial translocation and maintaining intestinal health (Schneider et al., 2000; Xie and Ye, 2013). Wu et al. (2022) adopted an intermittent oro-esophageal tube feeding (IOE) scheme for elderly Alzheimer’s patients with eating difficulties, implementing a nasogastric feeding tube protocol (Nousiainen et al., 2006). This method allows the patient to be fed in a short time and the continuous infusion of nutrients through the nasogastric tube ensures the direct absorption of nutrients from the intestine. This can meet the body’s normal protein requirements without the need for repeated intubation, which helps maintain the integrity of the gastrointestinal mucosal cell function and structure, strengthens the gastrointestinal mechanical and immune barriers, and prevents the translocation of intestinal endotoxins and bacteria. The results showed that after two months of intervention, the numbers of Bifidobacterium and Lactobacillus in the observation group were higher than those in the control group, while the numbers of Clostridium perfringens, Enterobacteriaceae, and Enterococci were lower than those in the control group, indicating that IOE can effectively improve the symptoms of gut microbiota imbalance in patients (**Tables 3** , **4**).

**Table 3:** *In vitro* cell experiments on gut microbiota dysbiosis.

No.	Experimental Materials/Methods	Intervention	Changes in Gut Microbiota	Conclusion
Lisbeth N	Gut NK cells	Lactobacillus reuteri DSM12246	Lactobacillus strongly induced the production of IL-12 in DCs, and mature DCs induced a large amount of IFN-γ in NK cells.	The evidence that IFN-γ-induced LAB strains can inhibit the action of IFN-γ-induced strains while retaining NK cell-stimulating activity may represent a key mechanism for maintaining gut immune homeostasis.
Sichetti M	Macrophage cell line THP1	Lactobacillus rhamnosus LR32, Bifidobacterium lactis BL04, and Bifidobacterium longum BL05	The probiotic formulation significantly increased the production of the anti-inflammatory cytokine interleukin-10 (IL-10) in macrophages and reduced the secretion of major pro-inflammatory cytokines IL-1β and IL-6 by 70% and 80%, respectively.	The probiotic formulation was first identified as having the capability to favor the M2 phenotype of macrophages.
Kim CS	63 fecal samples from healthy elderly individuals	Bifidobacterium bifidum BGN4 and Bifidobacterium longum BORI	Metabolites involved in bile acid metabolism influenced by the gut microbiota significantly shifted in the probiotic group.	Probiotic intervention in healthy elderly individuals can increase gut microbiota-derived IPA levels, which exert neuroprotective effects by modulating inflammatory signaling in BV2 microglial cells.
Liu F	Pig colitis cell model	Krill Oil (KO)	KO significantly reduced LPS-induced expression of IL-1β and TNFα in human macrophages in a dose-dependent manner by modulating broad-spectrum signaling pathways such as NF-κB and NOD-like receptor signaling, and showed synergistic effects with COX2 and IKK2 inhibitors in attenuating inflammatory pathways.	KO inhibited Th1 immune responses and promoted M1/M2 macrophage polarization, demonstrating the anti-inflammatory properties of KO and its role in promoting the resolution of intestinal inflammation.
Sun L	Fecal samples from T2D patients	Metformin	In the Bacteroides genus, the abundance of multiple species decreased after metformin treatment, with the reduction of Bacteroides fragilis being the most significant.	Oral metformin treatment can regulate the gut microbiota and bile acid metabolism in T2D patients.

**Table 4:** *In vivo* animal experiments on gut microbiota dysbiosis.

No.	Animal Model	Intervention	Changes in Gut Microbiota	Physiological/Pathological Manifestations in Animals
Thakur BK	Mouse Mesenteric Lymph Nodes (MLN)	Lactobacillus casei Lbs2	Lactobacillus casei Lbs2 significantly inhibited the secretion of pro-inflammatory cytokines (TNF-α, IL-6) induced by lipopolysaccharides.	Live and heat-killed Lbs2 polarized Th0 cells to T regulatory (Treg) cells *in vitro*, increased the frequency of FoxP3 Treg cells in mesenteric lymph nodes (MLN), and alleviated the macroscopic and histopathological features of colitis in probiotic-fed mice.
Yang W	Mouse Intestinal CD4 T Cells	Clostridium	SCFAs induced IL-22 in antigen-specific T cells of the gut microbiota, promoting the development of regulatory T cells (Treg) and the production of IL-10 by CD4 T cells.	The production of IL-22 by intestinal immune cells is regulated by a mechanism mediated by aryl hydrocarbon receptors and HIF1α through short-chain fatty acids, protecting mice from intestinal inflammation.
Ang QY	Mouse	Ketogenic Diet (KD)	Ketone bodies inhibited the growth of Bifidobacterium, leading to reduced levels of gut Th17 cells associated with KD, which may also result in reduced adipose tissue.	The difference between a ketogenic diet and a high-fat diet is that they alter the gut microbiome to affect the levels of gut Th17 cells.
Snart J	Rat Cecal Cells	β-Glucan	The abundance of rRNAs from Lactobacillus in the cecal content of β-glucan-fed rats was higher than that in the cecal content of cellulose-fed animals.	The increase in the abundance of Lactobacillus rRNAs was due to the large presence of Lactobacillus in the cecum of β-glucan-fed rats compared to the cecum of cellulose-fed animals.
Wang J	Heart Failure Rat Gut Microbiota	Ginseng	Different doses of GN may further affect myocardial injury by regulating the abundance of gut microbiota with different functions.	Ginseng can regulate microbial populations, increasing the proportion of short-chain fatty acids and anti-inflammatory bacteria, while reducing the proportion of conditional pathogens associated with a diabetic phenotype. Beisner J
Beisner J	Mouse Paneth Cells	Inulin and Sodium Butyrate	Inulin and sodium butyrate induced antimicrobial expression in Paneth cells, while a high-fat and high-sugar diet reduced antimicrobial expression in Paneth cells.	In Paneth cells, antimicrobial peptides are stored in cytoplasmic granules, from which they can be released into the intestinal lumen, contributing to host defense in the gut.

#### 5.2.4 Other Western medicines

In recent years, as research into intestinal microecology has deepened, the application of Western medicine in treating gastrointestinal dysfunction in elderly populations has garnered significant attention. When addressing gastrointestinal dysfunction in elderly patients, it is imperative to consider their physiological characteristics and the potential side effects of medications to develop personalized treatment plans (Hoel et al., 2021). 1) Antibiotics: commonly prescribed antibiotics for treating gastrointestinal dysfunction caused by bacterial infections include Cefradine Dispersible Tablets, Amoxicillin Capsules, and Cefuroxime Axetil Tablets. These medications target and eliminate pathogenic bacteria in the intestines, thereby alleviating symptoms such as diarrhea and abdominal pain. However, prolonged use of antibiotics can result in intestinal flora imbalance (Song et al., 2025); therefore, a thorough evaluation of the patient’s condition is essential prior to initiating antibiotic therapy. In particular, elderly patients may experience diminished liver and kidney function, which can impair their ability to metabolize and clear drugs. Consequently, dosage adjustments or alternative drug selections may be necessary. 2) Anti-diarrheal medications: agents such as Montmorillonite Powder and Compound Diphenoxylate Tablets alleviate diarrhea symptoms by either reducing intestinal peristalsis or protecting the intestinal mucosa. Specifically, Montmorillonite Powder adsorbs harmful substances within the intestines, thereby mitigating irritation (Neerati, 2025), while Compound Diphenoxylate Tablets inhibit smooth muscle contractions in the intestines, prolonging food retention time and minimizing water loss, thus effectively controlling diarrhea. It is important to note that in cases of infection-induced diarrhea, the sole use of anti-diarrheal drugs should be avoided to prevent exacerbating the condition (Neerati, 2025). 3) Gastrointestinal motility enhancers: medications like Domperidone Capsules and Mosapride Citrate Tablets enhance gastrointestinal peristalsis, promoting digestion and absorption, and alleviating constipation. These agents improve bowel function by increasing intestinal motility (Aikawa et al., 2025). 4) Gastric acid regulators: drugs such as Omeprazole Enteric-Coated Tablets and Ranitidine Hydrochloride Capsules mitigate discomfort from excessive gastric acid by inhibiting acid secretion and protecting the gastric mucosa. These medications are particularly effective in treating symptoms of acid reflux and gastric ulcers (Neerati, 2025). In elderly patients, where changes in gastric acid secretion may occur, dosages of these drugs should be adjusted according to individual conditions.

## 6 Conclusion and perspective

Achieving “healthy aging” is imperative. Healthy longevity results from a combination of various factors, generally divided into congenital and acquired factors, with acquired factors playing a crucial role in human health and longevity. Researchers Frieson and Luciga, who studied a hundred centenarians in Finland, believe that the environment and lifestyle habits may account for 66% of the factors contributing to longevity. Therefore, it is widely considered that acquired factors have a more practical impact on human health and longevity, suggesting that even without favorable congenital conditions, efforts can still lead to extended longevity.

In recent years, with the development of the economy, society, and improvements in medical and health security conditions, human lifespan has gradually extended. However, centenarians remain extremely rare in the total population. Numerous factors prevent humans from reaching their natural lifespan, including various social, economic, disease-related, nutritional, genetic, environmental, and mental state factors.

In the aging process, the ecological balance of gut microbiota is vital for maintaining overall health, the factors influencing gut microbiota imbalance in the elderly population are diverse, primarily including physiological decline due to aging, changes in dietary structure, and the impact of physical activity. These factors collectively impact the gut microecology, leading to a disruption in microbial balance and subsequently causing a series of health issues. In terms of Western medical interventions, recent research has mainly focused on adjusting dietary structure, supplementing probiotics and prebiotics, and using enteral nutrition. By optimizing the diet and increasing the intake of dietary fiber, the growth of beneficial bacteria can be promoted, maintaining microbial balance. At the same time, the supplementation of probiotics and prebiotics has also been proven to improve the structure of gut microbiota and alleviate related symptoms. TCM, on the other hand, emphasizes holistic concepts and syndrome differentiation treatment, having unique advantages in intervening with gut microbiota dysbiosis. By using single herbs or compound formulas, TCM can regulate qi and blood, strengthen the spleen and stomach, thereby improving the gut environment and promoting microbial balance. Moreover, TCM pays attention to individual differences and comprehensive treatment, enabling the formulation of personalized treatment plans based on the specific conditions of patients. This review has some limitations: 1) although the Western and Chinese medical intervention strategies mentioned include some research and practice, they may not cover all relevant studies. Additionally, the effectiveness of certain intervention strategies may lack long-term, large-sample, randomized controlled studies to confirm their efficacy and safety. 2) Gut microbiota dysbiosis is not only related to gut health but also closely associated with the health of multiple systemic systems. However, this review may not have fully covered the interactions between gut microbiota and other systems (such as the nervous system and immune system). Future research should strengthen interdisciplinary collaboration to explore the impact and intervention strategies of gut microbiota dysbiosis from a more comprehensive perspective (Ogunjobi et al., 2024). 3) Although this review mentioned the impact of aging, dietary structure changes, and physical activity on gut microbiota, it did not delve into the detailed mechanisms of how these factors specifically act on gut microecology to disrupt microbial balance. Future research should investigate these mechanisms more deeply to formulate more precise intervention strategies. In summary, the factors influencing gut microbiota dysbiosis in the elderly are complex and diverse, with both Western and Chinese medicine having their own characteristics in interventions. Future research should further explore the interaction mechanisms between gut microbiota and the host, as well as more precise and effective intervention strategies, providing strong support for the health management of the elderly population.
